# Structures of designed armadillo-repeat proteins show propagation of inter-repeat interface effects

**DOI:** 10.1107/S2059798315023116

**Published:** 2016-01-01

**Authors:** Christian Reichen, Chaithanya Madhurantakam, Simon Hansen, Markus G. Grütter, Andreas Plückthun, Peer R. E. Mittl

**Affiliations:** a Department of Biochemistry, University of Zürich, Winterthurerstrasse 190, 8057 Zürich, Switzerland

**Keywords:** armadillo repeat, protein engineering, calcium binding, peptide binding, solenoid protein

## Abstract

The armadillo repeat serves as a scaffold for the development of modular peptide-recognition modules. In order to develop such a system, three crystal structures of designed armadillo-repeat proteins with third-generation N-caps (Y_III_-type), four or five internal repeats (M-type) and second-generation C-caps (A_II_-type) were determined at 1.8 Å (His-Y_III_M_4_A_II_), 2.0 Å (His-Y_III_M_5_A_II_) and 1.95 Å (Y_III_M_5_A_II_) resolution and compared with those of variants with third-generation C-caps. All constructs are full consensus designs in which the internal repeats have exactly the same sequence, and hence identical conformations of the internal repeats are expected. The N-cap and internal repeats M_1_ to M_3_ are indeed extremely similar, but the comparison reveals structural differences in internal repeats M_4_ and M_5_ and the C-cap. These differences are caused by long-range effects of the C-cap, contacting molecules in the crystal, and the intrinsic design of the repeat. Unfortunately, the rigid-body movement of the C-terminal part impairs the regular arrangement of internal repeats that forms the putative peptide-binding site. The second-generation C-cap improves the packing of buried residues and thereby the stability of the protein. These considerations are useful for future improvements of an armadillo-repeat-based peptide-recognition system.

## Abstract 2

Designed armadillo-repeat proteins are promising scaffolds for modular peptide-recognition systems. The crystal structures of His-Y_III_M_4_A_II_, His-Y_III_M_5_A_II_ and Y_III_M_5_A_II_ highlight structural heterogeneity in full-consensus designs and aid the improvement of future constructs.

## 1. Introduction   

**Figure 1:**
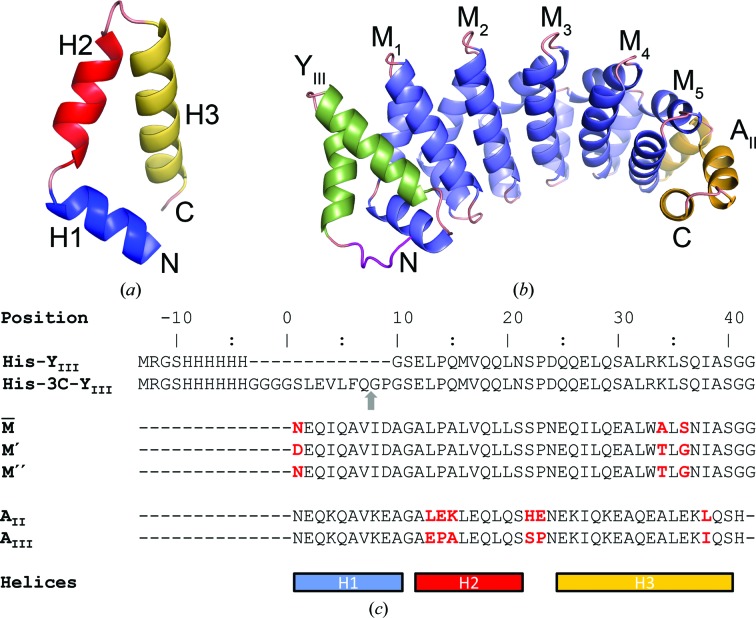
(*a*) The triangular spiral staircase arrangement of helices indicative of the armadillo repeat. (*b*) Ribbon diagram of His-Y_III_M_5_A_II_. The His_6_ tag, Y_III_-type, M-type and A_II_-type repeats are shown in magenta, green, blue and orange, respectively. (*c*) Sequence alignment of N-caps with and without a 3C protease cleavage site (the scissile bond is indicated by a grey arrow), internal repeats and C-­caps. Residues distinguishing different repeat versions are highlighted in red.

For the design of artificial peptide-binding modules, scaffolds with modular architectures are highly suitable. In particular, the armadillo repeat reveals structural properties that facilitate the design of peptide-binding modules on a rational basis (Andrade *et al.*, 2001 ▸; Kajander *et al.*, 2006 ▸; Boersma & Plückthun, 2011 ▸; Reichen, Hansen *et al.*, 2014 ▸). In natural armadillo-repeat proteins such as importin-α and β-catenin, each repeat comprises three α-helices that are assembled in a triangular spiral staircase arrangement. All repeats are fused into a single protein with an elongated hydrophobic core (Figs. 1 ▸ *a* and 1 ▸ *b*). They recognize their target peptides in extended β-sheet conformations with very regular binding topologies. The main chain of the peptide is bound in an antiparallel direction by conserved asparagine residues on the concave side of the armadillo-repeat protein (Huber *et al.*, 1997 ▸; Conti *et al.*, 1998 ▸; Kobe, 1999 ▸; Fontes *et al.*, 2003 ▸). Differences exist in side-chain preferences because the importin-α and β-catenin subfamilies recognize peptides with positively and negatively charged side chains, respectively (Conti & Kuriyan, 2000 ▸; Ishiyama *et al.*, 2010 ▸; Poy *et al.*, 2001 ▸).

It is the goal of this protein-engineering project to develop a stable full-consensus armadillo-repeat scaffold. Internal repeats with identical sequences are characteristic of full-consensus designs. Later, the internal repeats will be functional­ized to recognize different amino-acid side chains. The modularity of the design, which is imposed by the repetitive architecture, should enable us to generate artificial peptide-binding proteins with properties that are precisely tailored according to the length and sequence of the target peptide (Parmeggiani *et al.*, 2008 ▸; Reichen, Hansen *et al.*, 2014 ▸). Binding proteins with sequence-specific recognition properties for unstructured peptides should be of great interest in research and development because peptide–protein inter­actions represent 15–40% of all cellular interactions (Petsalaki *et al.*, 2009 ▸). Here, many protein–protein interaction scaffolds are unsuitable because they recognize targets based on surface-complementarity properties and thus require a folded counterpart. Conversely, many recognition modules used in intracellular signalling recognize only very short sequences and thus have very low affinity (Pawson & Nash, 2003 ▸). Indeed, specific peptide–protein interaction strategies are required to cope with the intrinsic flexibility of unstructured peptides (London *et al.*, 2010 ▸).

The first designed armadillo-repeat proteins (dArmRPs) were constructed using a consensus design approach based on 133 and 110 sequences from the importin-α and β-catenin subfamilies, respectively, in combination with structure-aided modifications of the hydrophobic core (Parmeggiani *et al.*, 2008 ▸). They possess the overall composition Y_*z*_M_*n*_A_*z*_, where Y, M and A represent the N-terminal, internal and C-terminal repeats, respectively. The subscripts denote the generation (version) count (*z*) and the number of internal repeats (*n*) in roman and arabic numbers, respectively. Since structure-based techniques are vital for this design approach, several structures of proteins from the Y_II_M_*n*_A_II_ and Y_III_M_*n*_A_III_ series have been determined. Initial crystal structures of dArmRPs with second-generation N- and C-caps revealed domain-swapped N-caps, suggesting that the Y_II_-type N-cap was unstable in solution. To improve the thermodynamic stability of the caps, nine and six mutations were inserted in the N- and C-caps, respectively. These modifications had complementary effects on the thermodynamic stability of the proteins. Introduction of the third-generation N-cap (Y_III_-type) increased the melting temperature by 4.5°C, but the modifications in the C-cap (A_III_-type) decreased it by 5.5°C (Madhurantakam *et al.*, 2012 ▸). The thermodynamic stabilities of dArmRPs that have so far been designed in this project have been summarized in Reichen, Hansen *et al.* (2014 ▸).

Although the initial crystal structures of His-Y_III_M_3_A_III_ and His-Y_III_M_3_A_II_ revealed monomeric proteins (Reichen, Madhurantakam *et al.*, 2014 ▸), later studies on Y_III_M_5_A_III_ (third-generation N-cap and C-cap) without an N-terminal His tag revealed domain-swapped N-caps and C-caps in the presence of calcium ions. However, domain swapping of Y_III_M_5_A_III_ was not observed either in the absence of calcium ions or in the presence of the His tag because the His tag prevented the unfolding of the N-cap by binding to the neighbouring His-Y_III_M_5_A_III_ molecule (Reichen, Madhurantakam *et al.*, 2014 ▸). To investigate the impact of the cap design on the structural parameters of dArmRPs, particularly in the absence of the His tag, we investigated the crystal structures of the more stable dArmRPs with third-generation N-caps and second-generation C-caps.

## 2. Materials and methods   

### 2.1. Cloning, protein expression and purification   

dArmRPs with cleavable and non­cleavable N-terminal His_6_ tags have been expressed and purified as described by Reichen, Madhurantakam *et al.* (2014 ▸) with the following modifications: vectors pPank and p148_3C were used for the expression of proteins with and without a cleavable His_6_ tag, respectively. The initial designs had non­cleavable His_6_ tags, but in order to facilitate the elimination of the purification tag, a 3C protease cleavage site was inserted between the His_6_ tag and the N-terminus of the N-cap. The amino-acid sequences of the internal and capping repeats are depicted in Fig. 1 ▸(*c*).

The proteins comprise third-generation N-caps, second-generation C-caps and four or five internal repeats. All three constructs are full-consensus designs, with internal repeats derived from the 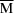-type internal repeat described in Alfarano *et al.* (2012 ▸). His-Y_III_M_4_A_II_ and Y_III_M_5_A_II_ contain M′-type internal repeats, whereas His-Y_III_M_5_A_II_ contains the M′′-type. In the M′′-type the aspartic acid at position 1, which was introduced to mimic a potential arginine-binding pocket, was mutated back to the consensus asparagine residue (for all sequences, see Fig. 1 ▸ *c*). To improve readability, we refer to M-type internal repeats throughout the text.

### 2.2. Crystallization and structure determination   

A Phoenix crystallization robot (Art Robbins Instruments) was used to set up sitting-drop vapour-diffusion experiments in 96-well Corning plates (Corning, New York, USA). Initial crystallization conditions were identified by sparse-matrix screens from Hampton Research (California) and Molecular Dimensions (Suffolk, England), and were later refined by grid screens in which the pH and the precipitant concentrations were varied simultaneously. To confirm the expected peptide-binding site, (KR)_5_ peptide was added to Y_III_M_5_A_II_ in a 1.5:1 molar ratio prior to crystallization. (KR)_5_ peptide was used for this experiment because the designed molecular surface of Y_III_M_5_A_II_ resembled the most conserved importin-α peptide-binding site, which recognizes with its core repeats (major and minor binding sites) positive dipeptide motifs composed of lysine and arginine residues. The rationale for this experiment is discussed in Reichen, Hansen *et al.* (2014 ▸). Protein solutions were mixed with reservoir solutions in 1:1, 1:2 or 2:1 ratios (200–300 nl final volume) and the mixtures were equilibrated against 50 µl reservoir solution at 4°C. Reservoir conditions are summarized in Table 1 ▸. After washing, the crystals in reservoir solutions supplemented with glycerol were flash-cooled in liquid nitrogen.

Data were collected on beamlines X06SA and X06DA at the Swiss Light Source (Paul Scherrer Institute, Villigen, Switzerland) using a Pilatus detector (Dectris, Baden, Switzerland) and a wavelength of 1.0 Å. Diffraction data were processed using *MOSFLM* (Leslie, 1992 ▸) and *SCALA* (Evans, 2006 ▸). Structures were solved by molecular replacement using *Phaser* (McCoy *et al.*, 2007 ▸) together with the following search models. For His-Y_III_M_4_A_II_ we used the structure of Y_III_M_3_A_II_ (PDB entry 4db6; Madhurantakam *et al.*, 2012 ▸). The refined His-Y_III_M_4_A_II_ structure was then used to solve the His-Y_III_M_5_A_II_ and finally the Y_III_M_5_A_II_ structures. The structures were refined using *PHENIX* (Adams *et al.*, 2010 ▸) and *REFMAC*5 (Murshudov *et al.*, 2011 ▸). For manual model building we used the program *Coot* (Emsley & Cowtan, 2004 ▸). The decrease in *R*_free_ suggested the use of different refinement strategies for His-Y_III_M_4_A_II_ and His-Y_III_M_5_A_II_. His-Y_III_M_4_A_II_ was refined without NCS restraints, whereas tight NCS restraints between chains *A*/*B* and *C*/*D* were applied for the refinement of His-Y_III_M_5_A_II_. Figures were prepared using *PyMOL* (DeLano, 2002 ▸). Metal ions were placed manually into strong difference electron-density peaks, taking into account the coordination geometry and the composition of the crystallization buffer. Calcium ions were validated by inspecting the anomalous difference map calculated with phases from the final structure. Water molecules were placed into well defined difference electron-density peaks at hydrogen-bond distance from the protein. No (KR)_5_ peptide was identified in the final electron-density map of Y_III_M_5_A_II_. Side-chain conformations were assigned according to the rotamer library of Dunbrack & Cohen (1997 ▸) as implemented in *Coot*.

## 3. Results and discussion   

### 3.1. Structures of His-Y_III_M_4_A_II_ and His-Y_III_M_5_A_II_   

The crystal structures of His-Y_III_M_4_A_II_ and His-Y_III_M_5_A_II_ were refined at 1.8 and 2.0 Å resolution, respectively. In both cases the asymmetric units contain tetramers with 222 point symmetry and very similar topologies. The quaternary structures of His-Y_III_M_4_A_II_ and His-Y_III_M_5_A_II_ are governed by calcium ions that connect neighbouring chains in a zipper-like manner and the His_6_ tag that binds to the supposed peptide-binding site, albeit in different orientations (see below).

**Figure 2:**
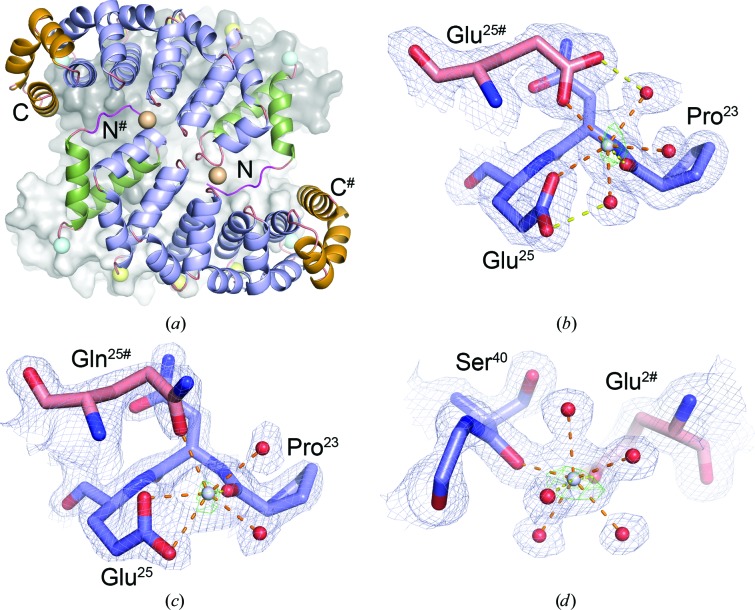
(*a*) The subunits of the His-Y_III_M_4_A_II_ tetramer are connected *via* calcium ions. Two chains are sketched as ribbons and coloured as described in Fig. 1 ▸(*b*). Two chains are shown as grey surfaces. Calcium ions are indicated as spheres. Calcium ions binding only to internal repeats are in yellow, those involving the N-cap in light blue and those at the twofold axis in salmon. (*b*) Calcium-binding site between internal repeats viewed along the axial direction (from the direction of Pro^23#^ O, which was omitted for clarity). Residues from different chains are shown as sticks with blue and salmon C atoms. Calcium ions and water molecules are depicted as grey and red spheres with reduced atomic radii, respectively. Polar interactions in the pentagonal plane involving the calcium ion are shown as dashed lines in orange. Additional interactions are in yellow. The 2*F*_o_ − *F*_c_ and anomalous difference electron-density maps are contoured at 1.3σ (light blue) and 4σ (green), respectively. (*c*) Calcium-binding site involving the N-cap. (*d*) Calcium-binding site at the twofold axis. Colour coding is as described for (*b*).

The His-Y_III_M_4_A_II_ tetramer contains 16 calcium ions. Five calcium ions connect two His-Y_III_M_4_A_II_ chains in an antiparallel orientation (Fig. 2 ▸ *a*). Considering the large size of this interface (average interface area of 1163 Å^2^) there are relatively few direct hydrogen bonds, and most interactions are made *via* calcium ions in the loops between helices H2 and H3. The coordination number of each calcium ion in His-Y_III_M_4_A_II_ is seven, which agrees very well with the statistical analysis of calcium-coordination geometry in protein and small-molecule complexes. Typically, the coordination number of calcium varies between six and eight, with an average length for coordination bonds of between 2.35 and 2.45 Å (Katz *et al.*, 1996 ▸). In His-Y_III_M_4_A_II_ the coordination geometry of calcium differs among ions that are bound to internal or capping repeats.

Ca^2+^ ions that bind to internal repeats are contacted by Pro^23^ O and Glu^25^ OE1 from two symmetry-related chains (superscripts indicate the position in the repeat as indicated in Fig. 1 ▸ *c*) and three water molecules (Fig. 2 ▸ *b*). Here, Glu^25^ contributes one coordination bond (Glu^25^ OE1–Ca distance 2.5 Å). In contrast, calcium ions that bind between an internal repeat and the N-cap are contacted by two water molecules, two O atoms from Glu^25^ (Glu^25^ OE1–Ca distance of 2.5 Å and Glu^25^ OE2–Ca distance of 3.0 Å), Gln^25^ OE1 and Pro^23^ O (Fig. 2 ▸ *c*). Thus, the replacement of glutamic acid at position 25 by glutamine in the N-cap displaces one water molecule and allows Glu^25^ to serve as a bidentate ligand. This observation agrees well with previous data on the statistics of calcium binding, in which it was shown that bidentate binding of carboxylate groups to calcium is particularly prevalent if the coordination number is greater than six (Katz *et al.*, 1996 ▸). In contrast to many natural calcium-binding sites, where all coordination bonds are approximately equal in length, the His-Y_III_M_4_A_II_ calcium-binding sites are distorted. In His-Y_III_M_4_A_II_ the axial calcium–ligand distances are shorter than the equatorial distances (axial distances 2.1–2.2 Å; equatorial distances 2.4–3.0 Å) and the Glu^25^ OE2–Ca bonding distances differ significantly from the average coordination bond length. The second coordination bond of Glu^25^ is longer, because the carboxylate group is rotated away from the Ca^2+^ ion. In contrast to natural calcium-binding sites that have evolved over time, the His-Y_III_M_4_A_II_ calcium-binding sites are distorted because they are artificial and are therefore less perfect. Besides these zipper-like Ca^2+^ ions bound to the N-termini of H3 helices, four well defined calcium ions additionally bind close to the twofold axes. These Ca^2+^ ions also show pentagonal-bipyramidal coordination spheres involving the Ser^40^ carbonyl O atom, the Glu^2^ side chain and five water molecules (Fig. 2 ▸ *d*). Furthermore, there are two weakly occupied calcium-binding sites involved in crystal contacts.

**Figure 3:**
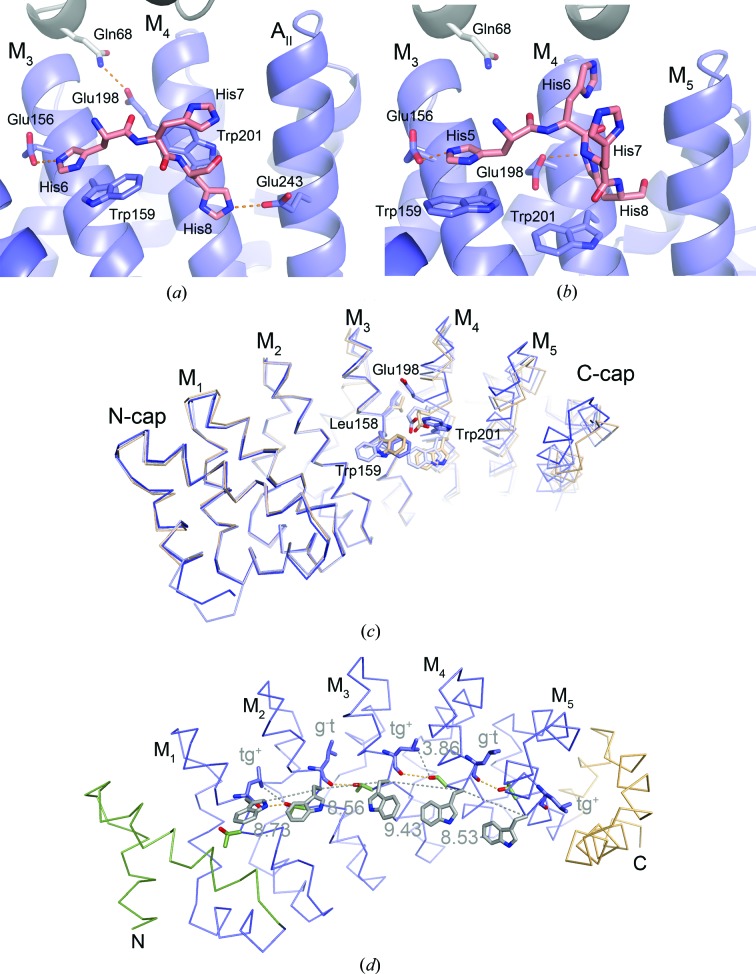
Interface between internal repeats M_3_ and M_4_ in chain *C* of His-Y_III_M_4_A_II_ (*a*) and His-Y_III_M_5_A_II_ (*b*). The dArmRPs are shown in blue and grey and the His_6_ tag with salmon C atoms. (*c*) Superposition based on the N-cap and internal repeats M_1_–M_3_ of His-Y_III_M_5_A_II_ chain *A* (dark blue), His-Y_III_M_5_A_II_ chain *C* (light blue) and Y_III_M_5_A_II_ (orange). Residues at the M_3_–M_4_ interface are labelled. (*d*) C^α^ trace of Y_III_M_5_A_II_ coloured in green (N-cap), blue (internal repeats) and orange (C-cap). The Leu^32^, Trp^33^ and Thr^34^ side chains are shown as sticks in blue, grey and green, respectively. Hydrogen bonds and general distances are shown as orange and grey dotted lines, respectively. Distances and conformations of Leu^32^ side chains are indicated (tg^+^, *trans*/*gauche* ^+^; g^−^t, *gauche* ^−^/*trans*).

The His-Y_III_M_4_A_II_ tetramer is further stabilized by interactions between the N-terminal His_6_ tag and the supposed peptide-binding site. This contact is formed by His6, which interacts with Glu^30^ and Trp^33^ (Glu156 and Trp159) from the third internal repeat, and His8, which interacts with Trp^33^ (Trp201) from the fourth internal repeat and Glu^33^ (Glu243) from the C-cap (Fig. 3 ▸ *a*). Besides the salt bridges between histidine and glutamic acid side chains, the aromatic stacking interaction between His6 and Trp^33^ might contribute significant binding energy because the spatial orientation of side chains seen here is frequently found in protein structures (cluster 4 of His–Trp interactions in the atlas of protein side-chain interactions; Singh & Thornton, 1992 ▸). Since all four chains of His-Y_III_M_4_A_II_ are very similar (r.m.s.d. of 0.28 Å for residues 14–246) these interactions are equivalent in all four subunits of the crystallographic tetramer.

In contrast to this, the crystallographic tetramer of His-Y_III_M_5_A_II_ is less symmetric. Here, chains *A*/*B* and *C*/*D* are pairwise identical (r.m.s.d. of 0.05 Å), whereas an r.m.s.d. of 0.85 Å for the comparison between pairs (*e.g.* chain *A* with *D*) suggests substantial differences. Furthermore, His-Y_III_M_5_A_II_ chains *A*/*B* are more similar to His-Y_III_M_4_A_II_ (r.m.s.d. of 0.72 Å for the superposition of residues 14–210 on the equivalent residues from His-Y_III_M_4_A_II_) than chains *C*/*D* (r.m.s.d. of 1.17 Å). These differences are caused by different contacts within the tetramer. In chains *C*/*D* of His-Y_III_M_5_A_II_ the side chain of Glu198 interacts with His8 from chain *D*/*C* (Fig. 3 ▸ *b*), whereas in chains *A*/*B* the side chain of Glu198 intercalates between internal repeats 3 and 4 and forms a hydrogen bond to the side chain of Gln68 from chains *B*/*A* (similar to the interaction shown in Fig. 3 ▸ *a* for His-Y_III_M_4_A_II_). As a consequence of this asymmetry, two calcium ions close to the twofold axis, which are present in all four chains of His-Y_III_M_4_A_II_ (Fig. 2 ▸ *d*), are only present in His-Y_III_M_5_A_II_ chains *A*/*B* and are absent from chains C/*D*.

### 3.2. Structure of Y_III_M_5_A_II_ without His tag   

The structure of Y_III_M_5_A_II_ without His tag was determined in the absence of calcium ions and refined at 1.95 Å resolution. This structure is most similar to chains *C*/*D* of His-Y_III_M_5_A_II_ (r.m.s.d.s of 1.14 and 0.60 Å for C^α^ atoms of residues 14–288 of chains *A*/*B* and *C*/*D*, respectively). These differences are a consequence of a rigid-body movement of the C-terminal repeats (internal repeats M_4_ and M_5_ and the C-cap). A superposition of Y_III_M_5_A_II_ on His-Y_III_M_5_A_II_ based on the N-cap and internal repeats M_1_–M_3_ (residues 14–168) reveals that this part is very similar in all chains. However, in this superposition the C-terminal repeats of Y_III_M_5_A_II_ match nicely with the C-terminal repeats of His-Y_III_M_5_A_II_ chains *C*/*D*, but they are shifted towards M_3_ in chains *A*/*B* (1.4 Å shift of Trp201 CA towards Leu158 CA). This movement can be described as an 8° rotation around an axis that runs parallel to the stacking direction of the C-terminal part and is probably a consequence of different side-chain conformations of Leu158, Trp159, Glu198 and Trp201 at the interface between M_3_ and M_4_ (Fig. 3 ▸ *c*). The structures of His-Y_III_M_4_A_II_ and Y_III_M_5_A_II_ represent extreme cases that are most different. In His-Y_III_M_5_A_II_ these differences are combined into a single structure. His-Y_III_M_5_A_II_ chains *A*/*B* and *C*/*D* represent the conformations seen in His-Y_III_M_4_A_II_ (all chains) and Y_III_M_5_A_II_ (all chains), respectively. Similar structural plasticity has been observed previously for the comparison of β-catenin crystallized in two different crystal forms. For β-catenin the C-terminal repeats were rotated 11.5° around an axis that runs approximately parallel to the axis of the superhelix (Huber *et al.*, 1997 ▸).

Thus, dArmRPs with second-generation C-caps and third-generation N-caps possess substantial flexibility, particularly for the side chains of Glu^30^, Leu^32^ and Trp^33^ (equivalent to Glu156, Leu158 and Trp159 in repeat M_3_ and Glu198, Leu200 and Trp201 in repeat M_4_). Experimental structural data for importin-α in complex with nuclear localization sequence (NLS) peptides (Conti *et al.*, 1998 ▸) and modelling studies on dArmRPs–peptide complexes (Reichen, Hansen *et al.*, 2014 ▸) indicate that the superhelix parameters and the conformations of Glu^30^ and Trp^33^, which also participate in binding the His_6_ tag as outlined above, are important structural features for proper binding of the target peptide. In a first approximation, the curvature of the peptide-binding site can be described by the distances of C^α^ atoms at position 33. In the major NLS peptide-binding site of importin-α (PDB entry 1bk6; Conti *et al.*, 1998 ▸) the distance between C^α^ atoms of adjacent Trp^33^ residues (*e.g.* Trp153, Trp195 and Trp237 in repeats 1–3) varies between 8.6 and 8.8 Å. In Y_III_M_5_A_II_ the average distance between these atoms is 8.82 ± 0.39 Å. However, in Y_III_M_5_A_II_ the spread between Trp^33^ C^α^-atom distances is extremely large, with the largest distance observed between repeats M_3_ and M_4_ (the distances between Trp159 CA and Trp201 CA are 9.42 Å in chain *A* and 9.43 Å in chain *B*). This distance is probably too large for binding the target peptide in the desired conformation and this mismatch is located almost at the centre of the putative peptide-binding site. It is possible that this mismatch is responsible for the fact that the (KR)_5_ peptide was not observed in the electron-density map, although it was present during crystallization. Interestingly, the rigid-body movement of the C-terminal part as seen in His-Y_III_M_4_A_II_ (all chains) and His-Y_III_M_5_A_II_ (chains *C*/*D*) brings this value to the other extreme. Here, the distance of Trp^33^ C^α^ atoms between repeats M_3_ and M_4_ is 8.14 ± 0.06 Å, which might be too short for proper binding.

Although Y_III_M_5_A_II_ is considered to be a full consensus design regarding the sequence of internal repeats, the internal repeats are not identical in terms of structure. These differences can be exerted either by different lattice contacts (Figs. 3 ▸ *a* and 3 ▸ *b*) or by improper design, which prevents the internal repeats from obtaining a unique conformation throughout the protein. Different distances between adjacent repeats are probably the result of both effects. In particular, the side-chain conformations of buried residues in the hydrophobic core, such as Ile^27^, Leu^32^, Thr^34^, Gly^36^ and Ile^38^, mediate the contacts between adjacent repeats. In the structure of Y_III_M_5_A_II_ the side-chain conformations of Thr^34^, Ile^38^ and of course Gly^36^ are invariant. The side chain of Thr^34^ cross-links internal repeats by forming hydrogen bonds to the main-chain carbonyl groups of Leu^32^ and Glu^30^ from adjacent repeats. The side chain of Ile^27^ adopts mainly *gauche* ^−^/*trans* conformations, whereas the side chain of Leu^32^ alternates between *trans*/*gauche* ^+^ and *gauche* ^−^/*trans* (Fig. 3 ▸ *d*).

This alternation suggests that a uniform conformation of Leu^32^ is impossible. In the interface between M_3_ and M_4_ of Y_III_M_5_A_II_, where we observe the largest distance between Trp^33^ C^α^ atoms, Leu158 CD1 (Leu^32^ in M_3_) and Thr202 OG1 (Thr^34^ in M_4_) are at van der Waals distances (3.86 and 3.97 Å in chains *A* and *B*) because the Leu158 side chain adopts a *trans*/*gauche* ^+^ conformation. Therefore, steric hindrance between Leu158 and Thr202 might be responsible for increasing the distance between Trp^33^ C^α^ atoms and for the failure to obtain a dArmRP–peptide complex structure. To adopt a Trp^33^ C^α^ distance which is similar to the values seen in the major binding site of importin-α, Thr202 OG1 would have to move closer to Leu158, but this approach would require a *gauche* ^−^/*trans* conformation of the Leu158 side chain. Of course, surface-exposed side chains (such as Trp^33^ and Glu^30^) also adopt different rotamers, but it can be assumed that these differences affect inter-repeat distances to a minor extent because the environments of surface-exposed side chains are usually less densely packed than the environments of buried side chains. However, some side-chain conformations of buried and surface-exposed residues are coupled. For example, the conformation of Trp^33^ is linked to the conformation of Leu^32^ in the preceding repeat. In repeats M_1_ and M_3_ Leu^32^ adopts *trans*/*gauche* ^+^ conformations and Trp^33^ in repeats M_2_ and M_4_ is *trans*/+90°, whereas in repeats M_2_ and M_4_ Leu^32^ is *gauche* ^−^/*trans* and Trp^33^ adopts *trans*/−105° conformations in repeats M_3_ and M_5_ (Fig. 3 ▸ *d*). Only Trp243 in chain *B* deviates from this general observation.

### 3.3. Comparison of dArmRPs with second-generation and third-generation C-caps   

The crystal structures of Y_III_M_5_A_III_ with and without a His_6_ tag and third-generation C-caps have been published recently (Reichen, Madhurantakam *et al.*, 2014 ▸). Y_III_M_5_A_III_ without a His_6_ tag but crystallized in the presence of calcium revealed domain-swapped N- and C-caps. Since Y_III_M_5_A_II_ without a His_6_ tag and a second-generation C-cap did not crystallize in the presence of calcium, it remains unclear whether the redesign of the C-cap was responsible for calcium-induced domain swapping.

**Figure 4:**
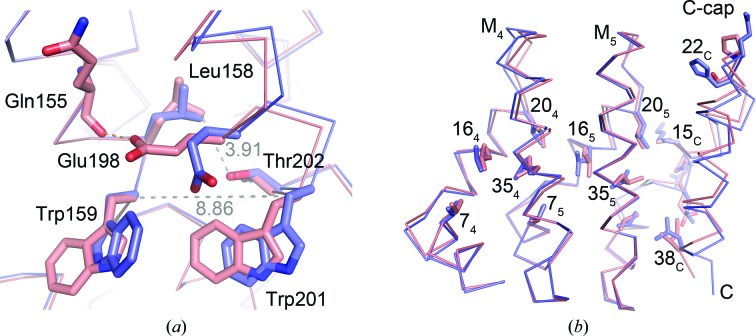
Superposition of Y_III_M_5_A_III_ (third-generation C-cap; PDB entry 4plq; salmon) on Y_III_M_5_A_II_ (second-generation C-cap; blue). (*a*) Residues at the M_3_–M_4_ interface. General distances and hydrogen bonds are shown as grey and orange dotted lines, respectively. Distance values refer to Y_III_M_5_A_III_. The superposition is based on all C^α^ atoms from M_3_. (*b*) Residues at the M_5_–C-cap interface. Numbers refer to positions in the repeat (Fig. 1 ▸ *c*), with subscripts indicating the internal repeat number or the C-cap. Side chains of all residues that differ between Y_II_ and Y_III_ and some residues from the hydrophobic core are shown in stick representation. The superposition is based on all C^α^ atoms from M_5_.

Interestingly, Y_III_M_5_A_III_ also shows an extended distance between Trp^33^ C^α^ atoms of internal repeats M_3_ and M_4_ (distance between Trp159 CA and Trp201 CA of 8.86 Å), a short distance between Thr202 OG1 and Leu158 CD2 of 3.91 Å and no electron density for the (KR)_5_ peptide, although it was present during crystallization (Reichen, Madhurantakam *et al.*, 2014 ▸). On the other hand, Leu158 shows the *gauche* ^−^/*trans* side-chain conformation, which is *trans*/*gauche* ^+^ in Y_III_M_5_A_II_, probably because Glu198 forms an additional hydrogen bond to Gln155 O (Fig. 4 ▸ *a*).

For dArmRPs with three internal repeats it was shown that the redesign of the C-cap (from A_II_ to A_III_) decreases the melting temperature by 5.5°C (Madhurantakam *et al.*, 2012 ▸), and a domain-swapped C-cap was observed for Y_III_M_5_A_III_ (Reichen, Madhurantakam *et al.*, 2014 ▸). Both observations suggest that Y_III_M_5_A_III_ is less stable than Y_III_M_5_A_II_. A superposition of Y_III_M_5_A_III_ (PDB entry 4plq) and Y_III_M_5_A_II_ based on the last internal repeat suggests that this destabilization might be owing to subtle rearrangements in the hydrophobic core between internal repeats M_4_ and M_5_ and the C-cap. Three out of six mutations that were introduced at the C-cap are solvent-exposed and do not seem to have a significant effect on the structure. However, Lys^15^→Ala, His^22^→Ser and Leu^38^→Ile mutations cause a gentle rearrangement of the C-cap (Fig. 4 ▸ *b*). This rearrangement has implications for the packing of side chains in the hydrophobic core. In the more stable Y_III_M_5_A_II_ structure the side chains of Leu^16^, Leu^20^ and Val^7^ adopt a uniform distribution of side-chain rotamers in all repeats. Val^7^ adopts a *trans* conformation. Leu^16^ and Leu^20^ are always *gauche* ^−^/*trans*. In Y_III_M_5_A_III_ this crystal-like arrangement is perturbed by the C-cap. In Y_III_M_5_A_III_ the side chains of Leu^16^, Leu^20^ and Val^7^ adopt the same conformations as in Y_III_M_5_A_II_ only in the N-terminal part, whereas in the C-terminal part their conformations are clearly different. For Leu^32^ the situation is inverted. In Y_III_M_5_A_III_ the rotamer distribution of Leu^32^ is uniform, whereas in Y_III_M_5_A_II_ alternating Leu^32^ conformations are observed (Fig. 3 ▸ *d*). Uniform distributions of rotamers are frequently observed in polypeptides with very high thermodynamic stabilities, such as amyloid fibrils (Nelson *et al.*, 2005 ▸) and β-helix proteins (Schulz & Ficner, 2011 ▸). Therefore, it can be assumed that the uniform distribution of side-chain rotamers is related to the stability of dArmRPs and *vice versa*. On the other hand, the deterioration of uniformity, as caused by the third-generation C-cap, is linked to destabilization of the protein.

In conclusion, this detailed investigation of the different versions of dArmRPs has shown that small differences in packing between repeats, notably between internal repeats and the caps, can make the protein susceptible to perturbations caused by crystal contacts and ions used in crystallization, indicating a lack of rigidity. This leads to a surprising long-range effect of changes in the C-cap and helps to explain the astonishing observation that a full-consensus design does not necessarily generate a unique repeat conformation. Although the internal repeats are chemically absolutely identical, their conformations lack uniformity. The current analysis suggests that future improvements of an armadillo-repeat-based peptide-recognition system will have to take three considerations into account. (i) In particular, the deletion of the His tag seems to be crucial for liberating the presumed peptide-binding site. (ii) The second-generation C-cap presented here seems to be superior to the third-generation C-cap, which was initially believed to be more advanced. (iii) The choice of amino acids at the inter-repeat interface, particularly at positions 27, 32 and 34, should be reconsidered because the side chains at these positions show substantial conformational heterogeneity.

## Supplementary Material

PDB reference: His-Y_III_M_5_A_II_, 4v3o

PDB reference: His-Y_III_M_4_A_II_, 4v3q

PDB reference: Y_III_M_4_A_II_, 4v3r

## Figures and Tables

**Table 1:** Data and refinement statistics Values in parentheses are for the highest resolution shell.

Structure	His-Y_III_M_4_A_II_	His-Y_III_M_5_A_II_	Y_III_M_5_A_II_
PDB code	4v3q	4v3o	4v3r
Data statistics			
Crystallization condition	25% PEG 2000 MME, 0.2 *M* calcium acetate, 0.1 *M* sodium acetate pH 5.5	15% PEG 4000, 0.2 *M* calcium acetate, 0.1 *M* sodium acetate pH 5.5	30% PEG 4000, 0.2 *M* magnesium chloride, 0.1 *M* Tris–HCl pH 8.5
Space group	*P*3_2_	*P*4_1_	*I*4
No. of molecules in asymmetric unit	4	4	2
Unit-cell parameters			
*a* = *b* (Å)	96.50	102.59	129.91
*c* (Å)	96.34	111.11	70.20
α = β (°)	90	90	90
γ (°)	120	90	90
Resolution (Å)	1.80 (1.91–1.80)	2.00 (2.11–2.00)	1.95 (2.06–1.95)
*R*_merge_ (%)	9.1 (88.6)	10.0 (75.0)	8.8 (47.6)
No. of observations	744192 (120009)	601165 (75390)	107908 (15424)
No. of unique reflections	93024 (15191)	76669 (10657)	41831 (6089)
〈*I*/σ(*I*)〉	12.6 (2.3)	12.2 (2.6)	7.5 (2.0)
Completeness (%)	100 (100)	94.3 (94.3)	98.1 (98.2)
Refinement statistics			
Resolution (Å)	96.34–1.80	111.11–2.00	91.86–1.95
*R*_cryst_ (%)	18.9	16.8	17.3
*R*_free_ (%)	23.6	22.4	22.9
*B* factors			
Wilson *B* (Å^2^)	27.0	28.7	21.4
Mean *B* value (Å^2^)	35.5	35.2	23.2
R.m.s.d. from ideal values			
Bond lengths (Å)	0.018	0.017	0.017
Bond angles (°)	1.83	1.71	1.72
Total No. of atoms			
Protein	7487	8618	4243
Water	654	767	419
Metal ions	16	12	3
Ligands	2	1	0
Ramachandran plot			
Favoured (%)	98.81	99.02	100.00
Allowed (%)	1.19	0.98	0.00
Outliers (%)	0.00	0.00	0.00
